# *Toxoplasma gondii*-Induced Activation of EGFR Prevents Autophagy Protein-Mediated Killing of the Parasite

**DOI:** 10.1371/journal.ppat.1003809

**Published:** 2013-12-19

**Authors:** Luis Muniz-Feliciano, Jennifer Van Grol, Jose-Andres C. Portillo, Lloyd Liew, Bing Liu, Cathleen R. Carlin, Vern B. Carruthers, Stephen Matthews, Carlos S. Subauste

**Affiliations:** 1 Department of Pathology, Case Western Reserve University, Cleveland, Ohio, United States of America; 2 Division of Infectious Diseases and HIV Medicine, Department of Medicine, Case Western Reserve University School of Medicine, Cleveland, Ohio, United States of America; 3 Division of Molecular Biosciences, Imperial College London, London, United Kingdom; 4 Department of Molecular Biology and Microbiology, Case Western Reserve University School of Medicine, Cleveland, Ohio, United States of America; 5 Department of Microbiology and Immunology, University of Michigan Medical School, Ann Arbor, Michigan, United States of America; 6 Department of Ophthalmology and Visual Sciences, Case Western Reserve University, Cleveland, Ohio, United States of America; Cornell University, United States of America

## Abstract

*Toxoplasma gondii* resides in an intracellular compartment (parasitophorous vacuole) that excludes transmembrane molecules required for endosome - lysosome recruitment. Thus, the parasite survives by avoiding lysosomal degradation. However, autophagy can re-route the parasitophorous vacuole to the lysosomes and cause parasite killing. This raises the possibility that *T. gondii* may deploy a strategy to prevent autophagic targeting to maintain the non-fusogenic nature of the vacuole. We report that *T. gondii* activated EGFR in endothelial cells, retinal pigment epithelial cells and microglia. Blockade of EGFR or its downstream molecule, Akt, caused targeting of the parasite by LC3^+^ structures, vacuole-lysosomal fusion, lysosomal degradation and killing of the parasite that were dependent on the autophagy proteins Atg7 and Beclin 1. Disassembly of GPCR or inhibition of metalloproteinases did not prevent EGFR-Akt activation. *T. gondii* micronemal proteins (MICs) containing EGF domains (EGF-MICs; MIC3 and MIC6) appeared to promote EGFR activation. Parasites defective in EGF-MICs (MIC1 ko, deficient in MIC1 and secretion of MIC6; MIC3 ko, deficient in MIC3; and MIC1-3 ko, deficient in MIC1, MIC3 and secretion of MIC6) caused impaired EGFR-Akt activation and recombinant EGF-MICs (MIC3 and MIC6) caused EGFR-Akt activation. In cells treated with autophagy stimulators (CD154, rapamycin) EGFR signaling inhibited LC3 accumulation around the parasite. Moreover, increased LC3 accumulation and parasite killing were noted in CD154-activated cells infected with MIC1-3 ko parasites. Finally, recombinant MIC3 and MIC6 inhibited parasite killing triggered by CD154 particularly against MIC1-3 ko parasites. Thus, our findings identified EGFR activation as a strategy used by *T. gondii* to maintain the non-fusogenic nature of the parasitophorous vacuole and suggest that EGF-MICs have a novel role in affecting signaling in host cells to promote parasite survival.

## Author Summary

*Toxoplasma gondii* resides in a parasitophorous vacuole that excludes transmembrane proteins required for recruitment of endosomes and lysosomes and thus, does not follow the path of classical lysosomal degradation. However, the non-fusogenic nature of the vacuole can be reverted when autophagy, a pathway to lysosomal degradation, is upregulated through the immune system or pharmacologically. Maintenance of the non-fusogenic nature of the vacuole is central to parasite survival. Thus, in addition to preventing degradation through a classical lysosomal pathway, *T. gondii* may also deploy strategies to prevent constitutive levels of autophagy from targeting the pathogen and causing its lysosomal degradation. We report that *T. gondii* accomplishes this task by causing EGFR activation in host cells. In cells that were not subjected to immune or pharmacologic upregulation of autophagy, blockade of EGFR resulted in parasite encasing by structures that expressed the autophagy protein LC3, vacuole-lysosomal fusion and autophagy protein-dependent killing of the parasite. Moreover, EGFR signaling also impaired targeting of the parasite by LC3^+^ structures in cells treated with stimulators of autophagy. Studies with *T. gondii* deficient in EGF domain containing-micronemal proteins (EGF-MICs) and recombinant EGF-MICs support the concept that these parasite adhesins contribute to EGFR activation.

## Introduction

*Toxoplasma gondii* is an obligate intracellular protozoan parasite that infects around a third of the human population worldwide. *T. gondii* is of clinical importance because it causes encephalitis in immunocompromised individuals and retino-choroiditis in immunocompetent and immunosuppressed patients. *T. gondii* can also cause congenital infection that may result in cerebral and ocular disease. Tachyzoites of *T. gondii* infect virtually any nucleated cell through active invasion. This process is dependent on the parasite actin-myosin motor and sequential secretion of proteins from micronemes and rhoptries, specialized organelles present in the apical end of the parasite [1]. Once secreted, *T. gondii* micronemal proteins (MICs) are expressed at the parasite surface membrane and they interact with host cell receptors [2]. MICs contain adhesive domains such as type I thrombospondin repeats, apple domains, EGF repeats and integrin A domains [3], [4]. The connection between transmembrane MICs to the actin-myosin motor (glideosome) of the parasite together with the binding of host cell receptors by MICs is considered to enable the organism to penetrate host cells [5], [6]. Following the release of MICs, rhoptries secrete rhoptry neck proteins (RONs) that are critical for the formation of a structure called the moving junction (MJ) [7], [8]. The MJ anchors the parasite to the host cell while the parasite penetrates it. The MJ is also believed to function as a sieve that excludes host type I transmembrane proteins from entering the PV membrane (PVM) [8], [9]. The end result is the formation of a parasitophorous vacuole that is devoid of host proteins required for recruitment of endosomes and lysosomes [10].

*T. gondii* cannot withstand the lysosomal environment. Thus, the non-fusogenic nature of the PV is critical since it allows the parasite to survive and replicate. The immune system can deprive the parasite from this niche by disrupting the PVM through the effects of IFN-γ/Immunity related GTPases (IRG) [11], [12] and by making the PV fusogenic through the effects of CD40 ligation [13]–[15]. CD40 re-routes the PV to the lysosomes through the autophagy machinery [13]–[15].

Autophagy is a conserved cellular mechanism of lysosomal degradation. During autophagy, portions of the cytosol or organelles are encircled by an isolation membrane [16]. The expansion of the isolation membrane results in the formation of a double membrane structure called autophagosome that delivers its contents to the lysosomes for degradation [16]. Autophagy is recognized as a mechanism stimulated by innate and adaptive immune mechanisms to degrade numerous intracellular pathogens [17]. However, various bacteria and viruses have evolved mechanisms to prevent autophagic degradation by targeting autophagy proteins to avoid recognition by the autophagy machinery or prevent the initiation and maturation of autophagosomes [18]–[24]. Much less is known regarding whether pathogens manipulate signaling cascades that regulate autophagy to prevent their degradation. HIV-1 envelope can activate the negative regulator of autophagy mTOR and it has been proposed that this would prevent lysosomal degradation of virions [25]. Bioinformatic analysis of human THP-1 cells infected with *Mycobacterium tuberculosis* suggested that the pathogen activates host signaling cascades that impair autophagy [26].

The highly successful nature of *T. gondii* as a pathogen together with evidence that the autophagy pathway can trigger lysosomal killing of the pathogen raise the possibility that *T. gondii* prevents autophagic targeting of the PV to maintain the non-fusogenic nature of the PV. Moreover, approximately 25–35% of various CD40^+^ cells subjected to CD40 ligation are unable to kill *T. gondii* further suggesting that the parasite may utilize mechanism(s) to prevent induction of autophagic killing. Here we report that maintenance of the non-fusogenic nature of the PV requires *T. gondii*-induced activation of EGFR-Akt, a signaling cascade that prevents autophagy protein-dependent vacuole-lysosomal fusion, lysosomal degradation and killing of the parasite. Blockade of EGFR-Akt may prove of therapeutic benefit for toxoplasmosis since it is sufficient to induce killing of the parasite without the need for immune-induced activation of host cells.

## Results

### *T. gondii* induces rapid Akt activation in non-hematopoietic cells through phosphatidylinositol 3-kinase (PI3K)

**Figure 1:**
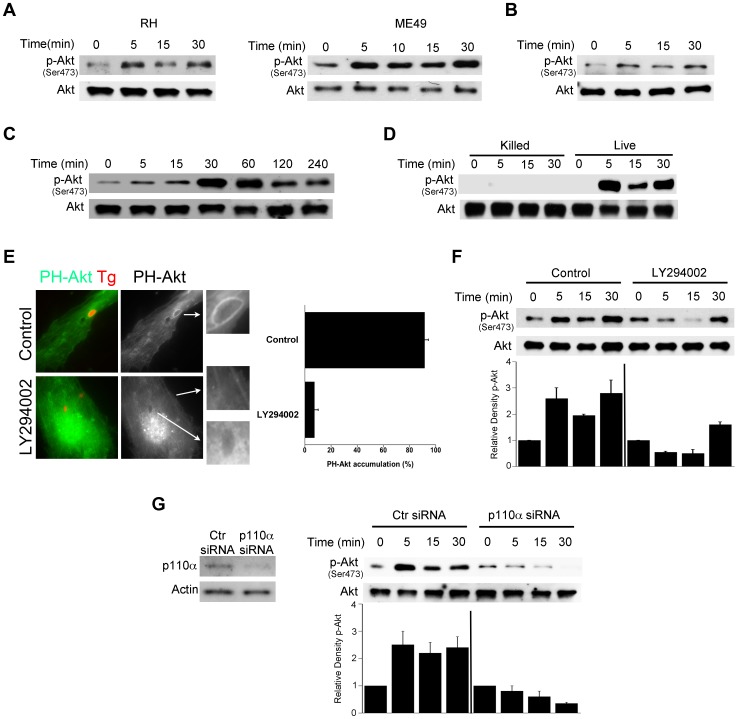
*T. gondii* induces Akt activation via PI3K signaling in non-hematopoietic cells. *A*, Primary HBMEC were challenged with type I (RH) or type II (ME49) strains of *T. gondii* for the indicated time points. Cell lysates were used to probe for total Akt and phospho-Ser473 Akt by immunoblot. *B*, *C*, Mouse endothelial cells (mHEVc; *B*) and human retinal pigment epithelial cells (RPE, *C*) were challenged with RH *T. gondii* followed by assessment of Akt phosphorylation. *D*, HBMEC were challenged with either live or killed *T. gondii*. Cell lysates were subject to immunoblotting as indicated. *E*, HBMEC were transiently transfected with a plasmid encoding PH-Akt-GFP. Cells treated with or without LY294002 (20 µM) for 1 h prior to challenge with *T. gondii*-RFP. Cells were examined by immunofluorescence at 5 min post-challenge to examine accumulation of PH-Akt-GFP around the parasites. *F*, HBMEC were incubated with LY294002 or vehicle for 1 h prior to challenge with *T. gondii*. Densitometry data represent means ± SEM of 4 experiments. A vertical line was inserted between densitometry data from control and LY294002-treated cells to indicate that band densities from infected cells treated with or without LY294002 are compared to bands from their respective uninfected cells, which were given an arbitrary number of 1. *G*, HBMEC were transiently transfected with control siRNA or PI3K p110α siRNA. Cells expressing either control siRNA or PI3K p110α siRNA were challenged with *T. gondii* 48 h after transfection. Cell lysates were subject to immunoblotting as indicated. Densitometry data are shown as above and represent means ± SEM of 3 experiments. Results shown are representative of 3–4 independent experiments.

We determined whether Akt is quickly activated by *T. gondii* during infection of various non-hematopoietic cells. Activation of Akt is a multistep process where phosphorylation of Serine 473 results in full activation of the molecule [27]. Primary human brain microvascular endothelial cells (HBMEC) were infected with either type I (RH) or type II (ME49) strains of *T. gondii* under conditions that caused synchronized infection. *T. gondii* infection resulted in an enhanced phosphorylation of Akt Serine 473 as assessed by immunoblot (Figure 1A). Similar results were obtained with a mouse endothelial cell line mHEVc (Figure 1B). *T. gondii* also caused Akt phosphorylation in a human retinal pigment epithelial (RPE) cell line, an effect that decreased at later time points post-infection (Figure 1C). We assessed whether viable parasites are required to induce activation of Akt. HBMEC were challenged with live or killed parasites followed by determination of Akt activation. Viable but not killed tachyzoites induced Akt phosphorylation (Figure 1D). Activation of phosphatidylinositol 3-kinase (PI3K) with resulting production of phosphatidylinositol 3,4,5 trisphosphate (PIP3) production is a major trigger of Akt activation [28]. The amino-terminal pleckstrin homology (PH) domain of Akt mediates recruitment of this molecule to plasma membrane containing increased PI(3,4,5)P3 or PI(3,4)P2 [29]. Indeed, the PH domain of Akt fused to GFP (PH-Akt-GFP) has been used as a probe to examine sites of PIP3 accumulation [30]. HBMEC were transiently transfected with a plasmid encoding PH-Akt-GFP followed by challenge with RH *T. gondii* that express cytoplasmic RFP (*T. gondii*-RFP). *T. gondii*-infected cells exhibited accumulation of PH-Akt-GFP around the parasite (Figure 1E). To examine the role of PI3K in this process, HBMEC were incubated with or without LY294002, a specific PI3K inhibitor, followed by challenge with the parasite. LY294002 did not affect the percentage of infected cells (not shown). Accumulation of PH-Akt-GFP around *T. gondii* was ablated by LY294002 (p<0.01) (Figure 1E). Moreover, incubation with LY294002 impaired the upregulation of Akt phosphorylation induced by *T. gondii*, especially in the earlier time points post-infection (Figure 1F). Similarly, Akt phosphorylation during *T. gondii* infection was impaired in HBMEC transfected with siRNA against the PI3K catalytic subunit p110α (Figure 1G). Taken together, these findings indicate that *T. gondii* induces rapid Akt activation in non-hematopoietic cells in a manner that is dependent on PI3K.

### Blockade of Akt induces accumulation of the autophagy protein LC3 around the parasite, vacuole-lysosome fusion and killing of *T. gondii* dependent on autophagy proteins

**Figure 2:**
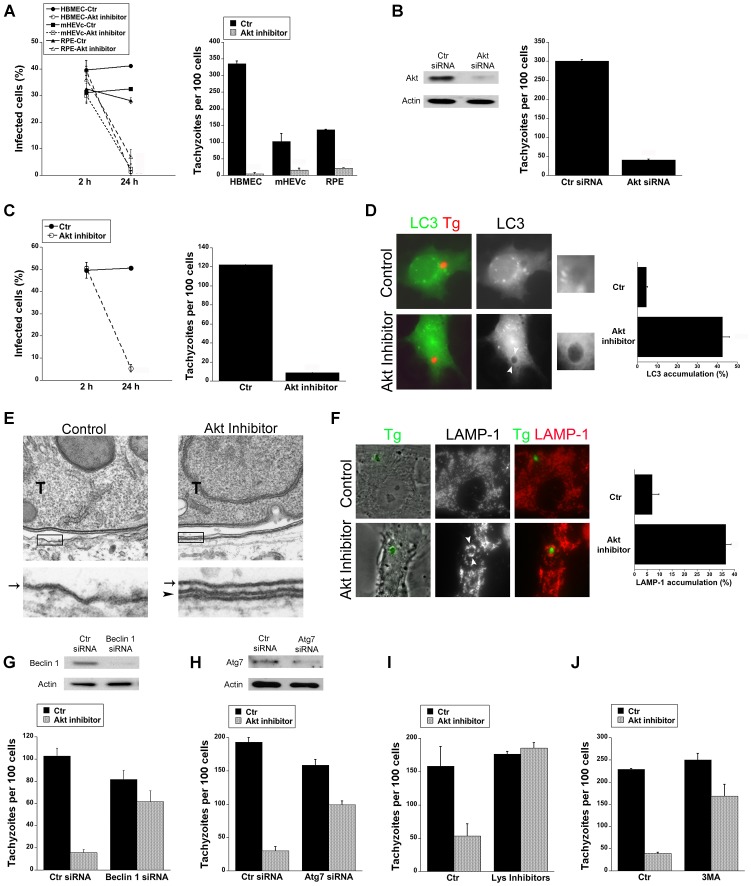
Blockade of Akt induces accumulation of the autophagy protein LC3 around the parasite, vacuole-lysosome fusion and killing of *T. gondii* dependent on the autophagy proteins. *A*, HBMEC, mHEVc and human RPE cells were incubated with or without Akt inhibitor IV (1.25 µM) for 1 h prior to challenge with *T. gondii*. Monolayers were examined by light microscopy 2 h and 24 h post-challenge. *B*, HBMEC were transfected with control siRNA or Akt siRNA. Cells were then challenged with *T. gondii* 48 h after transfection. Monolayers were examined microscopically 24 h post-challenge. *C*, RAW 264.7 were incubated with or without Akt inhibitor IV for 1 h prior to challenge with *T. gondii*. Monolayers were examined by light microscopy 2 h and 24 h post-challenge. *D*, mHEVc-LC3-EGFP cells were incubated with or without Akt inhibitor IV followed by challenge with *T. gondii*-RFP. Monolayers were examined by fluorescence microscopy 5 h post-challenge. Arrowheads indicate accumulation of LC3 around the parasite. *E*, HBMEC were treated with or without Akt inhibitor IV for 1 h prior to challenge with *T. gondii* (T) and then processed for electron microscopy at 5 h post-challenge. Images at the bottom represent magnification of the areas within the boxes. Arrow indicates the PVM; arrowhead indicates the double membrane structure around the vacuole. *F*, Control or Akt inhibitor IV-treated HBMEC were challenged with *T. gondii*-YFP. Expression of LAMP-1 was examined by fluorescent microscopy 8 h post-challenge. Arrowheads indicate accumulation of LAMP-1 around the parasite *G, H*, mHEVc cells were transfected with Beclin 1 siRNA (*G*), Atg7 siRNA (*H*) or control siRNA. After 48 h, cells were treated with or without Akt inhibitor IV for 1 h prior to challenge with *T. gondii*. Monolayers were examined by light microscopy at 24 h. *I*, mHEVc were treated with or without Akt inhibitor IV and infected with *T. gondii*. 1 h post infection cells were treated with or without leupeptin plus pepstatin (Lys inhibitors). Monolayers were examined microscopically 24 h post-challenge. *J*, Mouse microglia were incubated with or without Akt inhibitor IV. 3-methyl adenine (3MA; 10 mM) or vehicle were added 2 h post-challenge with *T. gondii*. Monolayers were examined microscopically 24 h post-challenge. Results are shown as the mean ± SEM and are representative of 3 independent experiments.

We performed studies to investigate whether blockade of Akt signaling promotes killing of *T. gondii*. HBMEC were incubated with or without Akt inhibitor IV followed by challenge with *T. gondii*. The percentage of infected cells at 2 hours and 24 hours post-challenge were determined. Akt inhibitor IV did not impair the percentage of infected cells at 2 h (Figure 2A). However, treatment with Akt inhibitor IV markedly reduced the percentage of infected cells at 24 h (p<0.01) (Figure 2A). Changes in the percentage of infected cells were not due to preferential cell loss in Akt inhibitor IV-treated cells since cell densities as determined with an eyepiece grid were similar in all experimental groups and inhibition of Akt did not induce a detectable increase in apoptosis of *T. gondii*-infected cells (not shown). Akt inhibitor IV not only induced a significant decrease in the numbers of parasites per 100 HBMEC at 24 h but it also caused a profound reduction in the numbers of *T. gondii*-containing vacuoles per 100 HBMEC (p<0.01) (Figure 2A, Figure S1A). Similar results were obtained with mouse endothelial cells (mHEVc; Figure 2A, Figure S1A) and human RPE cells (p<0.01) (Figure 2A, Figure S1A). Not only pharmacologic inhibition of Akt but also Akt knockdown in HBMEC reduced the parasite load and the number of *T. gondii*-containing vacuoles (p<0.01) (Figure 2B, Figure S1A). The vacuoles that persisted after Akt knockdown had similar numbers of parasites as those from control cells (Figure S1B). These results indicate that blockade of Akt caused parasite killing. *T. gondii* infection causes Akt activation in macrophages [31]. Similar to endothelial and epithelial cells, treatment with Akt inhibitor IV caused anti-*T. gondii* activity in the mouse macrophage line RAW 264.7 and in mouse microglia line BV-2 (p<0.01) (Figure 2C and not shown). These findings revealed an important role of Akt activation in promoting survival of *T. gondii* within host cells.

*T. gondii* survives within mammalian cells by avoiding delivery of the lysosomal contents into the parasitophorous vacuole [32]–[34]. Akt is a negative regulator of autophagy [35], a cellular mechanism that results in lysosomal degradation and killing of *T. gondii* [13]–[15]. First, we examined *T. gondii*-infected cells after Akt inhibition to determine the distribution of LC3, a protein associated with the autophagosome membrane. mHEVc-LC3-EGFP cells were treated with or without Akt inhibitor IV and challenged with *T. gondii*-RFP. Akt inhibitor IV led to significant accumulation of LC3 around the parasite (p<0.01) (Figure 2D). Electron microscopy studies were performed since a double membrane isolation membrane that encircles portions of cytoplasm or organelles is formed during autophagy [16]. Indeed, a double membrane structure was noted around the parasitophorous vacuole membrane in HBMEC treated with Akt inhibitor IV (Figure 2E). Next, we examined the effects of Akt inhibition on the distribution of the late endosomal/lysosomal molecule LAMP-1. Endothelial cells were incubated with or without Akt inhibitor IV, challenged with *T. gondii*-YFP followed by staining with anti-LAMP-1 mAb. Treatment with Akt inhibitor IV resulted in a remarkable increase in the percentage of parasites surrounded by LAMP-1 (p<0.01) (Figure 2F). To explore whether the killing of *T. gondii* during inhibition of Akt is dependent on the autophagy machinery, we examined the effects of knockdown of the autophagy proteins Beclin 1 or Atg7 on *T. gondii* survival. Transfection with Beclin 1 siRNA or Atg7 siRNA effectively diminished expression of Beclin 1 or Atg7 respectively (Figure 2G, H). Endothelial cells transfected with Beclin1 siRNA or Atg7 siRNA were incubated with or without Akt inhibitor and challenged with *T. gondii*. Cells transfected with Beclin1 siRNA (Figure 2G) or Atg7 siRNA (Figure 2H) were unable to control the parasite in the presence of the Akt inhibitor IV. Since autophagosomes deliver their contents to lysosomes for degradation, we examined the role of lysosomal degradation in killing of *T. gondii* utilizing the lysosomal protease inhibitors leupeptin and pepstatin. mHEVc and RPE cells were treated with or without Akt inhibitor IV and infected with *T. gondii*. 1 h post infection cells were treated with or without leupeptin plus pepstatin. Lysosomal protease inhibitors impaired the anti-*T. gondii* activity induced by Akt inhibition (p<0.05) (Figure 2I and not shown). Finally, the anti-*T. gondii* activity induced by Akt inhibitor IV in mouse microglia and human RPE cells was impaired by 3-methyl adenine, an inhibitor of autophagy (p<0.05) (Figure 2J and not shown). Taken together, these results indicate that *T. gondii*-induced Akt activation is critical to promote parasite survival because it prevents killing of *T. gondii* dependent on the autophagy pathway and lysosomal protease activity.

### *T. gondii* infection induces EGFR activation in mammalian cells that prevents autophagy pathway dependent killing of the parasite

**Figure 3:**
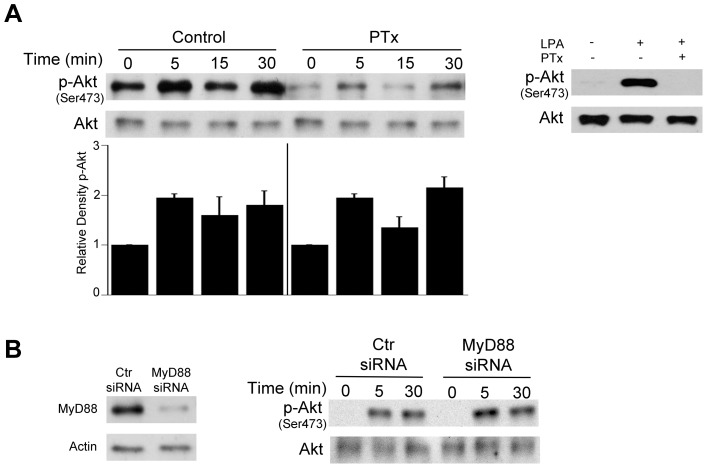
Role of G protein coupled receptors and MyD88 in *T. gondii*-induced Akt phosphorylation. *A*, HBMEC were treated with or without pertussis toxin (PTx; 100 ng/mL) for 4 h prior to challenge with *T. gondii*. Cell lysates were obtained at 5, 15 or 30 min post-incubation with *T. gondii* and used to examine total Akt and phospho-Akt serine 473 by immunoblot. Densitometry data represent means ± SEM of 3 experiments. A vertical line was inserted between densitometry data from control and PTx-treated cells to indicate that band densities from infected cells treated with or without PTx were compared to bands from their respective uninfected cells, which were given an arbitrary number of 1. HBMEC were also treated with or without LPA (10 µM) in the presence or absence of PTx. Cell lysates were obtained at 5 min and subjected to immunoblotting. *B*, HBMEC were transfected with MyD88 siRNA or control siRNA followed by challenge with *T gondii* after 48 h. Cell lysates were used to examine total Akt and phospho-serine 473 Akt by immunoblot. Results shown are representative of 3 independent experiments.

Akt activation classically occurs downstream of cell membrane receptors that include growth factor receptors, G protein-coupled receptor (GPCR) and TLR [36]. To examine the role of GPCR in Akt activation in non-hematopoietic cells, HBMEC were incubated with or without Pertussis toxin (PTx), an inhibitor of GPCR signaling, followed by challenge with *T. gondii* tachyzoites. PTx did not affect the initial percentage of infected cells (data not shown). Incubation with PTx decreased basal Akt phosphorylation. However, PTx did not prevent the increased Akt phosphorylation induced by *T. gondii* (Figure 3A) indicating that *T. gondii* can activate Akt independently of GPCR signaling. In contrast, PTx inhibited Akt activation induced by lysophosphatidic acid (LPA), a GPCR ligand [37] (Figure 3A). To examine the potential role of TLR signaling in Akt, MyD88 was knocked-down in HBMEC using siRNA. Knockdown of MyD88 did not affect *T. gondii*-induced Akt activation (Figure 3B). In contrast, as assessed by FACS, the ICAM-1 upregulation induced by LPS (1 µg/ml) in HBMEC was inhibited in cells transfected with MyD88 siRNA compared to those transfected with control siRNA (cMFI: Control siRNA = 10,682±1,053; MyD88 siRNA = 3,250±527; p<0.05). These studies indicate that GPCR and TLR are unlikely to play a major role in Akt phosphorylation induced by *T. gondii* in non-hematopoietic cells.

**Figure 4:**
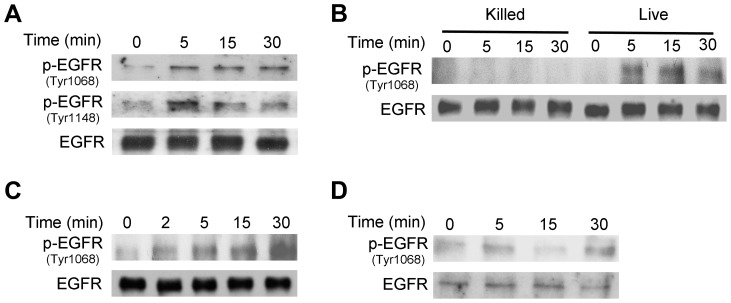
*T. gondii* infection induces EGFR activation in mammalian cells. *A*, HBMEC cells were challenged with RH *T. gondii* and cell lysates were obtained to probe for total EGFR, phospho-tyrosine 1068 EGFR and phospho- tyrosine 1148 EGFR by immunoblot. *B*, HBMEC were challenged with live vs killed tachyzoites. Total EGFR and phospho-tyrosine 1068 EGFR expression was examined by immunoblot. *C, D*, Human RPE cells (*C*) and mouse microglia (*D*) were challenged with RH *T. gondii* and total EGFR and phospho-tyrosine 1068 EGFR expression was examined by immunoblot. Results shown are representative of 3 independent experiments.

Relevant to the possibility of activation of growth factor receptors during *T. gondii*-host cell interaction is the fact that host cell invasion by *T. gondii* requires the secretion of parasite micronemal proteins (MICs) with the potential to activate such receptors [38]. MICs exist as multiprotein complexes, the most important being MIC1/4/6, MIC3/8, MIC2/M2AP, and a complex of the microneme protein TgAMA1 with rhoptry neck proteins RON2/RON4/RON6/RON8 [39]–[41]. MIC3, MIC6 and MIC8 have multiple domains with homology to EGF [42] and are therefore termed EGF-MICs. As an initial experiment, we examined whether *T. gondii* induces autophosphorylation at 2 major tyrosine residues of EGFR (1068 and 1148). HBMEC were incubated with RH *T. gondii* tachyzoites followed by determination of EGFR phosphorylation by immunoblot. *T. gondii* induced activation of EGFR, as indicated by phosphorylation of tyrosine residue 1068 (Figure 4A). Moreover, the parasite caused phosphorylation of tyrosine residue 1148, a site that appears to be phosphorylated only by ligand binding to EGFR [43] (Figure 4A). Similar results were found using the ME49 strain of *T. gondii* (not shown). Immunoblot analysis revealed that EGFR activation occurred in HBMEC upon challenge with viable but not killed parasites (Figure 4B). EGFR autophosphorylation was not only observed in endothelial cells but also in human RPE cells and mouse microglia incubated with *T. gondii* (Figure 4C, 4D). Thus, *T. gondii* causes EGFR activation in various mammalian cells.

**Figure 5:**
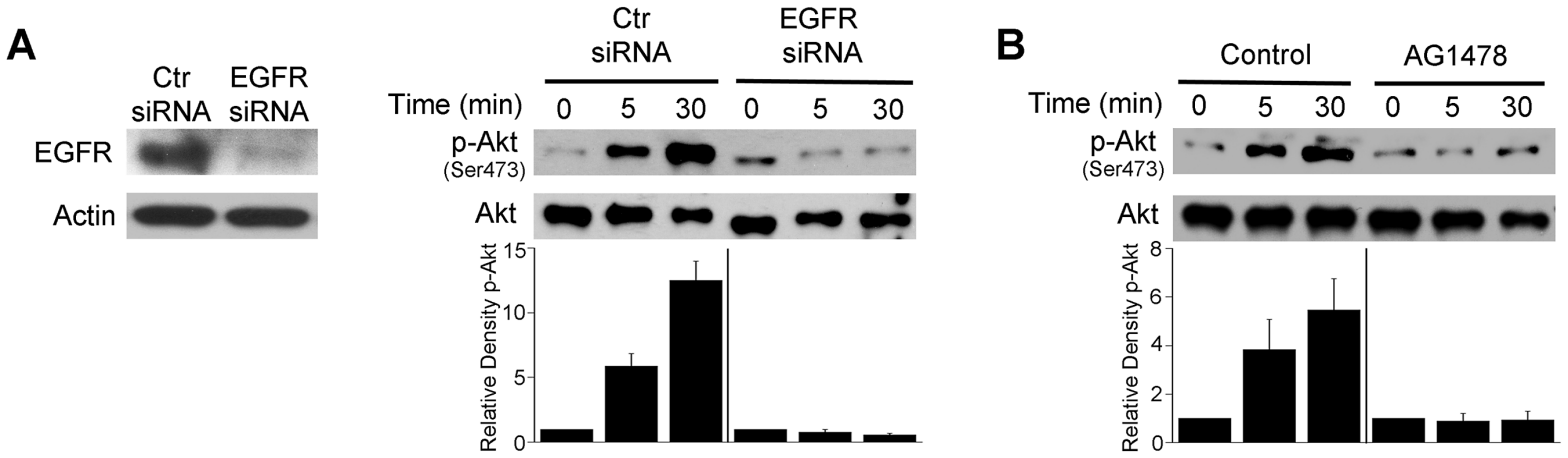
EGFR mediates Akt activation induced by *T. gondii*. *A*, mHEVc cells were transfected with control siRNA or EGFR siRNA. Expression of EGFR and actin were assessed by immunoblot 48 h post-transfection. mHEVc cells expressing either control siRNA or EGFR siRNA were challenged with *T. gondii*. Cell lysates were used to examine the expression total Akt or phospho-Ser473 Akt by immunoblot. *B*, Mouse microglia were treated with or without AG1478 (1 µM) 1 h prior to challenge with *T. gondii*. Cell lysates were used to examine the expression total Akt or phospho-Ser473 Akt by immunoblot. Densitometry data represent means ± SEM of 3 experiments. A vertical line was inserted between densitometry data from control siRNA and EGFR siRNA or control and AG1478-treated cells to indicate that band densities from infected control cells or infected cells subjected to EGFR blockade were compared to bands from their respective uninfected cells, which were given an arbitrary number of 1. Results shown are representative of 3 independent experiments.

Next, we examined whether EGFR signaling is involved in activation of Akt triggered by *T. gondii*. Endothelial cells were transiently transfected with a plasmid that encodes either control siRNA or EGFR siRNA followed by challenge with *T. gondii*. The efficiency of EGFR knockdown was confirmed by immunoblot (Figure 5A). EGFR knockdown ablated the ability of *T. gondii* to induce activation of Akt at all time points tested (Figure 5A). Next, we explored the role of EGFR signaling on Akt activation in professional phagocytes. Mouse microglia were treated with vehicle or AG1478, a pharmacological inhibitor of EGFR kinase activity, followed by challenge with *T. gondii*. Inhibition of EGFR kinase activity ablated parasite-induced Akt activation in mouse microglia (Figure 5B).

**Figure 6:**
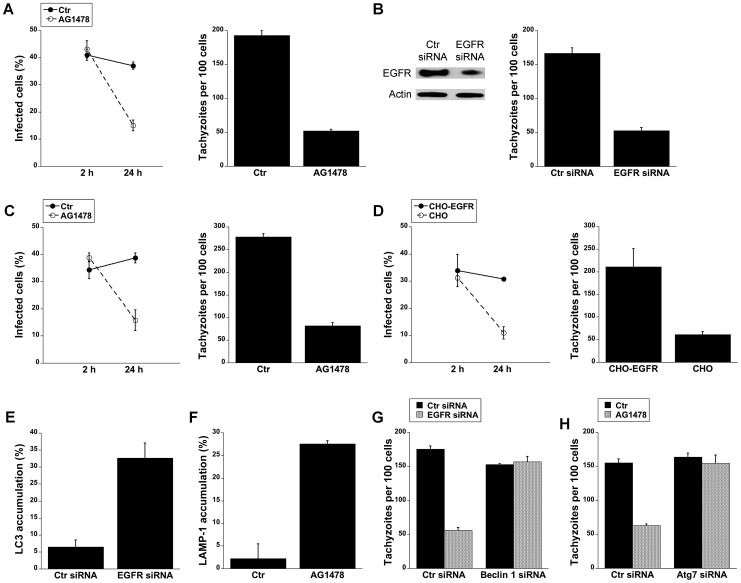
Blockade of EGFR induces accumulation of the autophagy protein LC3 around the parasite, vacuole-lysosome fusion and killing of *T. gondii* dependent on the autophagy proteins. *A*, HBMEC were incubated with AG1478 (1 µM) 1 h prior to challenge with *T. gondii*. Monolayers were examined by light microscopy at 2 and 24 h. *B*, Human RPE cells transfected with either EGFR or control siRNA were challenged with *T. gondii* followed by examination of monolayers by light microscopy at 24 h. *C*, Mouse bone marrow-derived macrophages were incubated with AG1478 and challenged with *T. gondii*. Monolayers were examined by light microscopy at 2 and 24 h. *D*, Parental CHO and CHO-EGFR cells were challenged with RH *T. gondii*. Monolayers were examined microscopically 2 h or 24 h post-challenge. *E*, mHEVc-LC3-EGFP cells transfected with control siRNA or EGFR siRNA were challenged with *T. gondii*-RFP. Monolayers were examined by fluorescence microscopy 5 h post-challenge to determine the percentage of endothelial cells with LC3 accumulation around the parasite. *F*, HBMEC cells treated with or without AG1478 were challenged with either *T. gondii*-YFP. Expression of LAMP-1 was examined by fluorescent microscopy 8 h post-challenge. The percentages of endothelial cells with LAMP-1 accumulation of around the parasite were determined. *G, H*, mHEVc cells transfected with Beclin1 siRNA (*G*) or Atg7 siRNA (*H*) were transfected with EGFR siRNA or treated with or without AG1478 followed by challenge with *T. gondii*. Monolayers were examined by light microscopy 24 h post-challenge. Results are shown as the mean ± SEM and are representative of 3 independent experiments.

We assessed whether EGFR activation affects *T. gondii* survival within host cells. HBMEC were treated with vehicle or AG1478 followed by challenge with *T. gondii*. While AG1478 did not affect the percentage of infected cells at 2 h, AG1478 caused a marked reduction in the percentage of infected cells 24 h post-challenge (p<0.05) (Figure 6A). In addition, there was a significant reduction in the numbers of parasites per 100 endothelial cells (p<0.01) (Figure 6A). Similar results were obtained whether HBMEC or human retinal endothelial cells were infected with RH or ME49 strains of *T. gondii* (not shown). The role of EGFR in affecting parasite survival was confirmed with a genetic approach since knockdown of EGFR in human RPE cells resulted in enhanced killing of *T. gondii* (p<0.01) (Figure 6B). Similar to the studies of blockade of Akt, inhibition of EGFR signaling not only reduced the percentages of infected cells but also caused a reduction in the numbers of vacuoles per 100 cells without affecting the numbers of parasites in the vacuoles that persisted after EGFR blockade (not shown). The effects of EGFR signaling inhibition were not restricted to non-hematopoietic cells since mouse bone marrow-derived macrophages also acquired anti-*T. gondii* activity when treated with AG1478 (p<0.05) (Figure 6C). To further explore the role of EGFR in the survival of *T. gondii*, we took a reverse approach and infected parental CHO cells, known to be EGFR null [44], and CHO cells expressing human EGFR (CHO-EGFR). A reduction in the percentage of infected cells and a reduction in parasite load at 24 h were observed in parental CHO cells compared to CHO-EGFR cells (p<0.05) (Figure 6D). These findings revealed an important role of EGFR in promoting Akt activation and *T. gondii* survival within host cells.

We investigated whether *T. gondii* killing induced by inhibition of EGFR is dependent on autophagy proteins. Knockdown of EGFR in mHEVc cells or treatment of these cells with AG1478 resulted in an enhanced accumulation of LC3 and LAMP-1 around the parasite (p<0.05) (Figure 6E and 6F). Moreover, silencing of Beclin 1 or Atg7 prevented induction of anti-*T. gondii* activity in endothelial cells subjected to EGFR knock-down or treated with AG1478 (p<0.01) (Figure 6G and 6H). Taken together, activation of EGFR signaling promoted survival of *T. gondii* within host cells by inhibiting autophagy protein-dependent killing of the parasite.

### *T. gondii* MICs can induce phosphorylation of EGFR and Akt in host cells

**Figure 7:**
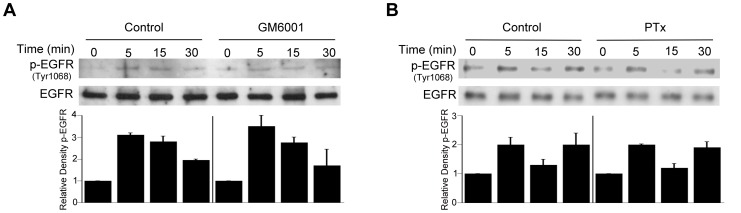
Role of metalloproteinases and G protein coupled receptors in *T. gondii*-induced EGFR phosphorylation. HBMEC were treated with or without GM6001 (10 µM) for 1 h prior to challenge with *T. gondii* (*A*) or with Pertussis Toxin (PTx; 100 ng/ml) for 4 h prior to parasite challenge (*B*). Cell lysates were used to examine total EGFR and phospho-tyrosine 1068 EGFR by immunoblot. Densitometry data represent means ± SEM of 3 experiments. A vertical line was inserted between densitometry data from control and GM6001- or PTX-treated cells to indicate that band densities from infected cells treated with or without these inhibitors are compared to bands from their respective uninfected cells, which were given an arbitrary number of 1. Results shown are representative of 3 independent experiments.

EGFR ligands exist as precursors transmembrane proteins that are shed from the plasma membrane by members of the ADAM (a disintegrin and metalloprotease) family of zinc-dependent metalloproteases [45]. This results in an autocrine or paracrine EGFR activation, a phenomenon that explains how proteins such GPCR activate EGFR [45]. We explored whether EGFR activation triggered by *T. gondii* could be due to this mechanism of autocrine/paracrine signaling. HBMEC were treated with GM6001, a broad spectrum ADAM inhibitor, followed by challenge with *T. gondii*. GM6001 did not affect the percentage of infected cells (data not shown) and did not prevent the ability of *T. gondii* to induce EGFR activation (Figure 7A). Moreover, EGFR phosphorylation after *T. gondii* infection took place despite incubation with PTx (Figure 7B). These findings suggest that ADAM- and GPCR-dependent EGFR activation do not play a major role in EGFR phosphorylation induced by *T. gondii*.

**Figure 8:**
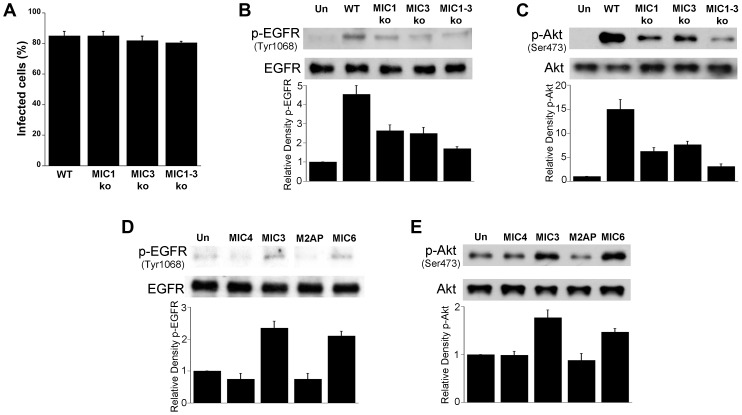
*T. gondii* micronemal proteins appear to induce EGFR and Akt activation. *A*, *B, C* HBMEC were challenged with ΔH_x_ (WT), MIC1 ko, MIC3 ko, MIC1-3 ko *T. gondii* at MOIs that yielded similar percentages of infected cells (*A*). Cell lysates were obtained and used to examine total EGFR and phospho-tyrosine 1068 EGFR (*B*) or total Akt and phospho-Ser 473 Akt (*C*) by immunoblot. HBMEC were incubated with 10 nM of recombinant MIC4, MIC3, M2AP or MIC6 for 15 minutes followed by examination of total EGFR and phospho-tyrosine 1068 EGFR (*D*) or total Akt and phospho-Ser473 (*E*) by immunoblot. Densitometry data represent means ± SEM of 4 experiments. Band densities from infected cells or cells treated with MICs were compared to bands from uninfected or untreated cells (Un), which were given an arbitrary number of 1. Results shown are representative of 4 independent experiments.

As stated above, MIC3, MIC6, MIC8 have multiple domains with homology to EGF [42]. MIC7 and MIC9 also express EGF-like domains but these MICs have poor or no expression in tachyzoites [42]. We examined the effect of deficiency of MICs on the ability to induce activation of EGFR and Akt. HBMEC were infected with wild type (WT), MIC1 ko (lacks MIC1, resulting in deficient secretion of MIC6 [46]), MIC3 ko (lacks MIC3), MIC1-3 ko (lacks MIC6 secretion and MIC3) parasites followed by determination of EGFR and Akt activation. These MIC ko parasites still express MIC8 (MIC8 deficiency results in parasites that are unable to infect mammalian cells). The multiplicity of infection was adjusted so that the initial percentages of infected HBMEC were similar for all strains of the parasite (Figure 8A). Compared to WT *T. gondii*, MIC1 ko and MIC3 ko parasites caused a partial reduction in EGFR and Akt phosphorylation (p<0.05) (Figure 8B, 8C). MIC1-3 ko parasites caused further decrease in EGFR and Akt phosphorylation compared to MIC1 ko and MIC3 ko parasites (p<0.05) (Figure 8B, 8C). However, even in cells infected with MIC1-3 ko parasites the reduction in EGFR and Akt phosphorylation was not complete. MIC1-3 ko parasites still express MIC8, a molecule that has EGF-like domains. We used conditional MIC8 knockout *T. gondii* previously generated using a tetracycline-inducible system to explore the potential role of MIC8 in signal activation [47]. Incubation of these parasites with anhydrotetracycline (ATc) results in almost complete ablation of MIC8 [47]. Parasites previously grown in the absence or presence of ATc were incubated with HBMEC. We could not detect an appreciable decrease in Akt phosphorylation in cells exposed to MIC8 deficient parasites (Figure S2). To further explore the role of MICs in the activation of EGFR and Akt, HBMEC were incubated with *Pichia pastoris*-derived MIC3. Although the EGF-like domains alone do not appear to promote the adhesion of MIC3 to mammalian cells [48], it was still possible that MIC3 could cause EGFR and Akt activation. Indeed, compared to recombinant MIC4 (a control that does not express EGF-like domains) incubation with recombinant MIC3 caused enhanced phosphorylation of EGFR and Akt in HBMEC (Figure 8D, 8E). Moreover, incubation with *E. coli*-derived MIC6 but not M2AP caused EGFR-Akt phosphorylation (Figure 8D, 8E). This response was unlikely to be mediated by LPS since M2AP and MIC6 preparations had similar concentrations of LPS (12 ng/ml and 12.4 ng/ml respectively). In addition, LPS at concentrations between 10–1,000 ng/ml failed to induce EGFR phosphorylation in HBMEC (not shown). Taken together, EGF-MICs (MIC3 and MIC6) can induce EGFR-Akt activation and parasites deficient on these MICs have diminished capacity to activate EGFR and Akt.

### EGFR signaling and MICs impair autophagic killing of *T. gondii*

**Figure 9:**
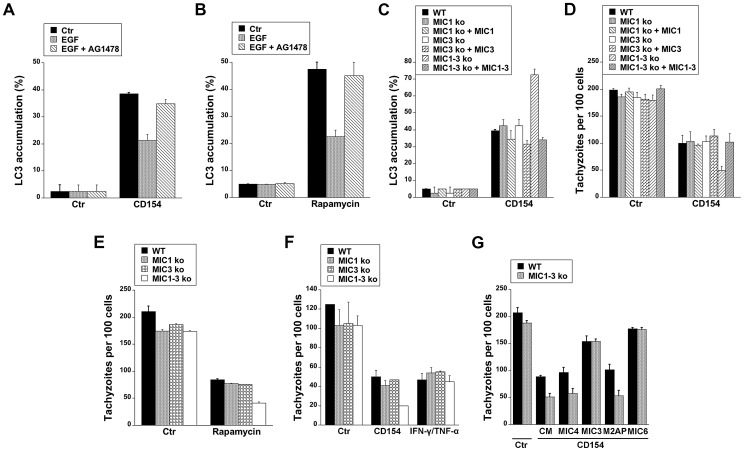
EGFR and *T. gondii* micronemal proteins modulate CD40-induced recruitment of LC3 around *T. gondii* and parasite killing. *A*, hmCD40 mHEVc expressing LC3-EGFP were treated with or without CD154 followed by challenge with *T. gondii*-RFP in the presence or absence of EGF (50 ng/ml) or AG1478 (1 µM). LC3 accumulation around *T. gondii* was assessed by immunofluorescence. *B*, mHEVc-LC3-EGFP cells were infected with *T. gondii*-RFP. Cells were treated with vehicle or rapamycin (1 µM) 2 h after challenge with *T. gondii* in the presence or absent of EGF or AG1478. Recruitment of LC3 around the parasite was examined at 5 h after challenge with *T. gondii*. *C*, hmCD40 mHEVc-LC3-EGFP cells were treated with or without CD154 followed by challenged with WT, MIC1 ko, MIC1 ko+MIC1, MIC3 ko, MIC3 ko+MIC3, MIC1-3 ko, MIC1-3 ko+MIC1-3 tachyzoites. LC3 accumulation around the parasite was examined by fluorescence microscopy 5 h post-challenge. *D*, hmCD40 mHEVc-LC3-EGFP treated with or without CD154 were challenged with WT, MIC1 ko, MIC1 ko+MIC1, MIC3 ko, MIC3 ko+MIC3, MIC1-3 ko or MIC1-3 ko+MIC1-3 tachyzoites. Parasite load was examined 24 h post-challenge. *E*, HBMEC cells were challenged with WT, MIC1 ko, MIC3 ko, MIC1-3 ko tachyzoites followed by treatment with vehicle or rapamycin. Parasite load was examined 24 h post-challenge. *F*, HBMEC treated with or without CD154 or IFN-γ/TNF-α were challenged with WT, MIC1 ko, MIC3 ko, MIC1-3 ko tachyzoites. Monolayers were examined microscopically at 24 h post-challenge. *G*, hmCD40 mHEVc cells treated with or without CD154 were infected with either WT or MIC1-3 ko tachyzoites in the presence of complete medium (CM) alone or medium plus MIC3, MIC4, M2AP or MIC6 (all 10 nM). Parasite load was determined microscopically 24 h post challenge. Results shown are representative of 3–4 independent experiments.

Cells stimulated with CD154 (CD40 ligand) exhibit accumulation of LC3 around *T. gondii* and killing that is dependent on autophagy proteins [13]–[15]. We examined whether targeting of the parasite by LC3^+^ structures in CD154-treated cells can be affected by EGFR signaling. Endothelial cells were treated with or without CD154 followed by challenge with *T. gondii* in the presence or absence of EGF. EGF did not affect the initial percentage of infected cells (not shown). As previously reported [15], CD154 caused accumulation of LC3 around *T. gondii* (Figure 9A). Targeting of parasites by LC3^+^ structures was inhibited in cells that were exposed to EGF (p<0.05) (Figure 9A), The effect of EGF was specific since addition of AG1478 to cells treated with EGF restored LC3 accumulation around *T. gondii* (Figure 9A). Similar results were obtained using rapamycin, a well-described stimulator of autophagy (Figure 9B). Next, we explored the role of MICs on the distribution of LC3^+^ structures in endothelial cells treated with CD154. Endothelial cells were treated with or without CD154 followed by challenge with WT, MIC1 ko, MIC3 ko, MIC1-3 ko and their respective complemented parasites. Infection with MIC1 ko, MIC3 ko or MIC1-3 ko parasites induces a partial decrease in EGFR-Akt activation (see Figures 8B, 8C). Indeed, in control endothelial cells (no CD154 treatment) there were no differences in the low level LC3 accumulation around the parasites (Figure 9C). After treatment with CD154, enhanced accumulation of LC3 around the parasites was similar in endothelial cells infected with WT, MIC1 ko or MIC3 ko parasites (Figure 9C). In contrast, cells infected with MIC1-3 ko parasites (the strain that was the weakest inducer of EGFR-Akt activation) exhibited a significant further increase in LC3 accumulation (p<0.05) (Figure 9C). These results were specific because the phenotype was lost in the complemented strain of *T. gondii* (MIC1-3 ko+MIC1-3) (Figure 9C). Examination of the parasite load revealed that the loads of MIC1 ko, MIC3 ko and MIC1-3 ko parasites were not significantly different from those of WT parasites in control endothelial cells (no CD154 treatment) (Figure 9D). When cells were treated with CD154, MIC1-3 ko *T. gondii* displayed increased susceptibility to CD154-induced anti-*T. gondii* activity (p<0.05) (Figure 9D). Similar to the studies of LC3 expression, the phenotype of MIC1-3 ko parasites was lost in the complemented strain (MIC1-3 ko+MIC1-3) (Figure 9D). Next, we examined whether increased killing of MIC1-3 ko parasites was observed in cells treated with another autophagy inducer (rapamycin) or in cells treated with IFN-γ, a cytokine that triggers anti-T. *gondii* activity independently of autophagic degradation [13]–[15]. Similar to CD154-stimulated cells, MIC1-3 ko parasites were more susceptible to rapamycin-induced killing (p<0.05) (Figure 9E). Moreover, in contrast to the results obtained with CD154-stimualtion, anti-*T. gondii* activity induced by IFN-γ/TNF-α was similar in all parasite strains tested including MIC1-3 ko *T. gondii* (Figure 9F). Finally, we explored the effects of recombinant MICs on CD154-induced killing of MIC1-3 ko *T. gondii*. In initial experiments, recombinant MICs did not affect the load of *T. gondii* in non-activated (control) endothelial cells or cells treated with IFN-γ/TNF-α (not shown). Next, control or CD154-activated endothelial cells were challenged with WT or MIC1-3 ko parasites in the presence of absence of recombinant MICs. Whereas treatment of endothelial cells with MIC4 and M2AP did not affect the load of WT or MIC1-3 ko parasites in CD154-activated cells, treatment with MIC3 or MIC6 inhibited CD154-induced *T. gondii* activity (p<0.05) (Figure 9G). Moreover, the phenotype of MIC1-3 ko parasites of increased susceptibility to CD154-mediated anti-*T. gondii* activity was lost in the presence of either MIC3 or MIC6 since the loads of WT and MIC1-3 ko parasites were no longer different in cells treated with these EGF-MICs (Figure 9G). Taken together, our findings indicate that EGFR, MIC3 and MIC6 negatively regulate autophagic killing of *T. gondii*.

## Discussion

Avoidance of lysosomal degradation is pivotal for the survival of numerous intracellular pathogens including *T. gondii*. Our studies indicate that, in addition to exclusion of type I transmembrane proteins from the PVM, *T. gondii* also activates EGFR-Akt signaling in the host cell to prevent targeting of the parasite by LC3^+^ structures and pathogen killing that is dependent on autophagy proteins and lysosomal protease activity. Thus, these studies identified EGFR-Akt signaling as a pathway critical for pathogen survival. In addition, they suggest that EGF-MICs may be involved in pathogen virulence not only by allowing parasite invasion of host cells but also by activating host cell signaling that counter-regulates autophagy.

Various bacteria and viruses encode virulence factors that impair the function of autophagy proteins and as a result, avoid their degradation via the autophagy pathway [18]–[24]. It has been suggested that HIV-1 and *M. tuberculosis* may prevent autophagic degradation by affecting signaling cascades that regulate the autophagy pathway [25], [26]. Our studies indicate that indeed a pathogen can act at the level of a regulatory pathway to avoid its degradation by the autophagy machinery. Relevant to our findings is the report that HIV-1 tat impairs autophagy by stimulating counter-regulatory cascades (Akt and STAT3), although these studies did not examine whether these pathways would prevent lysosomal degradation of the virions [49].

Our studies indicate that *T. gondii*-induced EGFR activation is a major event upstream of Akt phosphorylation in endothelial and RPE cells, a finding consistent with the important role of EGFR and other growth factor receptors as activators of Akt signaling [36], [50]. PI3K is a classical link between growth factor receptors and Akt activation. However, in contrast inhibition of EGFR signaling, the effect of PI3K inhibition on Akt activation appeared to be more transient. These findings may be explained by the fact that, besides PI3K, there are additional activators of Akt that might be engaged by growth factor receptors [51]. *T. gondii* has been reported to activate Akt in macrophages, a phenomenon that was inhibited by PTx [31]. Our studies indicate that EGFR also contributes to Akt activation in macrophages/microglia since the parasite caused EGFR autophosphorylation and inhibition of EGFR signaling impaired parasite-induced Akt activation. Moreover, not only activation of Akt but also activation of EGFR in endothelial cells, RPE cells and macrophages/microglia prevented killing of *T. gondii* dependent on autophagy proteins and lysosomal enzymes. The fact that Akt activation has been linked to inhibition of apoptosis of *T. gondii*-infected cells [31] raises the possibility that parasite-induced EGFR - Akt signaling may not only promote parasite survival by preserving the non-fusogenic nature of the PV but also by avoiding death of infected cells subjected to pro-apoptotic signals. While EGFR is a central mediator of Akt activation in the early stages after *T. gondii*, Akt phosphorylation has recently been reported at 24 h post-infection with the parasite [52]. This raises the possibility that *T. gondii* may also activate Akt through additional mechanisms besides parasite engagement of EGFR.

Although *T. gondii* causes EGFR - Akt activation and these signaling molecules have been shown to inhibit autophagy [35], [53], [54], *T. gondii* does not appear to prevent autophagosome formation in infected cells. Indeed, large LC3^+^ structures were readily detected within infected cells during early stages post-infection (see Figure 2D), a finding previously reported in host cells at 24 h post-infection [55]. Moreover, there is no decrease in the levels of LC3 II (the lipidated form of LC3 that associates with the autophagosome membrane) during the early stages of infection (Muniz-Feliciano and Subauste, unpublished observations). In fact, *T. gondii* has been reported to increase LC3 II levels and autophagosome formation in host cells at 24 h post-infection, presumably as an attempt to gain access to nutrients [55]. Our studies indicate that while global autophagy did not appear to be inhibited by *T. gondii*, engagement of EGFR impaired targeting of the PV by LC3^+^ structures. Future studies that identify how autophagosomes target the PV will likely shed light on the molecular mechanism by which EGFR - Akt diminish autophagic targeting of the parasite.

Various pathogens can target EGFR. *Pseudomonas aeruginosa* and *Helicobacter pylori* can cause EGFR phosphorylation that is mediated by the release of membrane-bound EGF ligands and transactivation of EGFR [56], [57]. *Klebsiella pneumonia* causes EGFR activation that appears to be dependent on bacterial capsule polysaccharide engagement of TLR4 and subsequent Src-dependent EGFR activation [58]. In addition, proteins from oncogenic viruses activate EGFR to mediate transformation [59]. Much less is known on whether microbial products can directly engage and activate EGFR. It has been suggested that *H. influenza* may activate EGFR through the presence of bacterial-derived molecules with EGF-like properties [60]. Uptake of Influenza A virus causes EGFR activation, a process that may be dependent on multivalent binding of hemagglutinin to sialic acids present on EGFR or ganglioside GM1 leading to aggregation of rafts, clustering of EGFR and its activation [61]. Our studies suggest that EGF-MICs play a role in mediating EGFR-Akt activation of host cells and prevention of parasite killing since: recombinant EGF-MICs (MIC3 and MIC6) induce EGFR-Akt activation while MICs that lack EGF domains do not cause appreciable phosphorylation of EGFR and Akt; EGFR signaling inhibits LC3 accumulation around *T. gondii*; parasites deficient in 2 EGF-MICs (MIC3 and MIC6: MIC1-3 ko parasites) cause markedly impaired EGFR-Akt activation and exhibit increased encasing by LC3^+^ structures as well as killing in cells treated with autophagy stimulators; MIC3 and MIC6 impair parasite killing mediated by the autophagy pathway.

It was interesting to note that MIC1-3 ko parasites are not targeted by LC3^+^ structures and are not more likely to be killed in unstimulated cells despite the markedly weakened EGFR-Akt signaling. MIC1-3 ko parasites only display increased susceptibility to autophagic targeting and killing when autophagy is stimulated by CD154 or rapamycin. Of relevance to our findings, other studies support the existence of signaling thresholds that need to be achieved in order for autophagy to take place [62], [63]. For example, in *Drosophila* both the Ret-like receptor tyrosine kinase Stitcher (Stit) and insulin receptor (InR) are required for cell growth and proliferation through the PI3K-I/TORC1 pathway in the wing disc [63]. A decrease in either Stit or InR signaling diminishes TORC1 activity and suppresses growth [63]. However, this decrease in TORC1 activity is not sufficient to trigger autophagy in the wing [63]. Autophagy only takes place when both Stit and InR are impaired [63]. It was proposed that the simultaneous inactivation of Stit and InR reduces PI3K-I activity and TORC1 signaling below a critically low level at which autophagy in the wing can no longer be prevented [63]. Given that the EGFR-Akt pathway inhibits autophagy by regulating TORC1 activity, a similar phenomenon could be at play in the case of *T. gondii* infection. The reduction in EGFR-Akt observed in cells infected with MIC1 ko or MIC3 ko parasites does not translate in increased autophagic killing of these parasites either in unstimulated cells or in cells treated with stimulators of autophagy. The further reduction in EGFR-Akt signaling observed in cells infected with MIC1-3 ko may still be sufficient to prevent autophagic killing in unstimulated cells but results in enhanced killing in cells treated with autophagy stimulators. Finally, further inhibition of EGFR-Akt signaling (by genetic or pharmacological approaches) triggers autophagic targeting of *T. gondii* even in unstimulated cells. Thus, our studies suggest that the effects of MIC deficiency on the levels of EGFR-Akt activation likely explain the differences in outcome observed after infection. Taken together, in addition to being key for invasion of host cells, EGF-MICs (MIC3 and MIC6) contribute to the induction of a signaling cascade within these cells that is required to avoid lysosomal degradation of the parasite.

While MIC1-3 ko parasite exhibited a marked defect in EGFR-Akt activation in host cells, phosphorylation of these molecules still took place. Although we cannot rule out a role of MIC8 in activation of this cascade, it appears that the residual ability of MIC1-3 ko parasites to activate EGFR-Akt may not be explained by their expression of MIC8 (an EGF-MIC). Conditional MIC8 ko parasites did not exhibit a noticeable defect in signal activation in host cells. These findings are likely explained by the fact that MIC8 ko parasites do not exhibit defects in attachment to host cells and they secrete MICs [47]. The presence of an additional mechanism of EGFR-Akt activation that normally cooperates with MIC-dependent EGFR signaling may explain why MIC1-3 ko *T. gondii* have residual capacity to activate the EGFR-Akt pathways.

*T. gondii* is very successful as a pathogen and utilizes various strategies to manipulate host cell signaling to ensure its survival [64]–[67]. Here we report that the parasite activates EGFR - Akt to maintain the non-fusogenic nature of PV a process that appears to be dependent at least in part on EGF-MICs. These findings may be of therapeutic relevance since various inhibitors of EGFR are being used for treatment of cancer. The fact that EGFR inhibition induced parasite killing in cells not treated with immune activators, raises the possibility that this approach may be effective even in immunocompromised hosts.

## Materials and Methods

### Mammalian cells

Primary human brain microvascular endothelial cells (HBMEC) were obtained from ScienCell Research Laboratories (Carlsbad, CA) and cultured in fibronectin-coated tissue culture flasks and basal medium supplemented with Endothelial Cell Growth Supplement (ECGS) and 5% fetal bovine serum (FBS) all from ScienCell. The mouse high endothelial venule cell line (mHEVc) (gift from Joan Cook-Mills, Northwestern University, Chicago, IL) and mHEVc cells stably expressing LC3-EGFP construct (mHEVc-LC3-EGFP) or hmCD40 plus LC3-EGFP (hmCD40 mHEVc-LC3-EGFP) [15] were cultured in DMEM plus 10% FBS (HyClone; Logan, UT). A human RPE cell line (ARPE-19; American Type Culture Collection, Manassas, VA), a mouse macrophage line (RAW 264.7) and mouse microglia line (BV-2) were cultured in DMEM plus 10% FBS. Mouse bone marrow-derived macrophages were obtained as described and cultured in DMEM plus 30% L929-conditioned medium, 10% FBS and 5% horse serum [68]. Parental Chinese Hamster Ovary (CHO) cells and CHO cells expressing human EGFR (CHO-EGFR) were cultured in MEM plus 10% FBS.

### *T. gondii* and infection

Experiments were conducted using tachyzoites of the RH strain of *T. gondii* (Type I strain), RH that express cytoplasmic YFP [69] or cytoplasmic DsRed (RFP) [69], tachyzoites of the ME49 (type II strain), transgenic parasites deficient in micronemal proteins MIC1 ko, MIC3 ko, MIC1-3 ko and the complemented strains (MIC1ko+MIC1, MIC3 ko+MIC3 and MIC1-3 ko+MIC1-3; gift from Maryse Lebrun, Universite de Montpellier 2, France) [39], as well as conditional MIC8 ko parasites (gift from Markus Meissner, University of Glasgow). Parasites were maintained in human foreskin fibroblasts following standard procedures [70]. In order to deplete MIC8, conditional MIC8 ko parasites were cultured in HFF in the presence of anhydrotetracycline (1 µg/ml) for 48 h. *T. gondii* tachyzoites were killed by incubation in 1% paraformaldehyde in PBS.

A potassium buffer shift was used to synchronize *T. gondii* invasion of serum-starved (0.1% FBS) mammalian cells as described [71]. Briefly, freshly egressed tachyzoites were resuspended in Endo buffer and incubated with cells for 20 minutes at 37°C. The Endo buffer was replaced for a low-potassium permissive medium to allow parasite invasion. In certain experiments, mammalian cells were incubated with Akt inhibitor IV (1.25 µM; EMD Millipore, Billerica, MA), PI3K inhibitor (LY294002; 20 µM; Sigma-Aldrich; St. Louis, MO), EGFR inhibitor (AG1478; 1 µM; EMD Millipore), a broad spectrum ADAM inhibitor (GM6001; 10 µM; EMD Millipore) (all 1 h prior to challenge with *T. gondii*), Pertussis Toxin (PTx; 100 ng/ml; EMD Millipore; 4 h prior to challenge), leupeptin (10 µM; EMD Millipore) and pepstatin (10 µM; EMD Millipore; both 1 h after challenge with *T. gondii*), 3-methyl adenine (3MA; 10 mM; Sigma Chemical) and rapamycin (1 µM; EMD Milipore; both 2 h after challenge with *T. gondii*) or vehicle. To induce CD40 signaling, mHEVc cells were treated with cell-free supernatants containing either multimeric human CD154 or a non-functional CD154 mutant [72] (T147N; both obtained from Dr. Richard Kornbluth, Univ. of California San Diego, current address Multimeric Biotherapeutics Inc., La Jolla, CA) for 18 h at 37°C as previously described [73] prior to challenge with parasites. Monolayers were fixed at indicated time points and stained with Diff-Quick (Dade Diagnostics, Aguada, Puerto Rico). The percentage of infected cells, the numbers of tachyzoites and vacuoles per 100 cells as well as the numbers of parasites per vacuole were determined by light microscopy by counting at least 200 cells or 200 vacuoles per monolayer as previously described [15].

### *T. gondii* proteins

For expression of MIC3 and MIC4 in *P. pastoris*, amplified DNA fragments were cloned into a pPICZα A vector (Invitrogen; Carlsbad, CA). The pPICZα A vector contains the *S. cervisiae* α-factor secretion signal that allows for the secretion of folded proteins from *P. pastoris*. Cells were grown in BMGY media, washed and resuspended in BMMY media for an initial OD600 of 20–40. The culture was then incubated in a 28°C incubator with vigorous shaking. The culture was then grown for 1–5 days depending on the optimal period of expression. Inhibition of glycosylation during culture required the addition of 20 µg/ml of tunicamycin. The supernatant is then passed through a HiTrap Q HF Column (GE Healthcare; Little Chalfont, UK). The eluted fraction was buffer exchanged into nickel-column binding buffer (50 mM Tric-HCl, pH 8.0, 50 mM NaCl). If needed further protein purification was achieved by a further nickel affinity step and gel filtration.

M2AP and MIC6 encompassing residues 87 to 197 (including EGF2 and EGF3 motifs) were generated using a pET32 Xa/LIC plasmid (Novagen, EMD Millipore) in the Origami (D3) *Escherichia coli* strain (Stratagene) [74], [75]. Expressions of the fusion protein was induced by adding 1 mM IPTG and harvested after overnight culture at 18°C. The cells were collected by centrifugation and lysed by French Press. The fusion protein incorporating a hexahistidine tag was purified by bench top chromatography using a nickel-nitrilotriacetic acid resin (QIAGEN). The fusion partner of protein was cleaved by factor Xa and removed by an additional chromatography step and the factor Xa was removed by agarose resins (Novagen). Protein samples were concentrated to 0.5 mM in 50 mM NaCl, 50 mM potassium phosphate and 5% D_2_O at pH 5.8. Endotoxin concentrations were similar in MIC3 and MIC4 as well as in M2AP and MIC6.

### Transfection

Cells were transiently transfected with a plasmid that encodes Akt-PH-GFP [76], human PI3K p110α siRNA [77], human Akt siRNA [78], mouse Beclin1 siRNA [79], mouse Atg7 siRNA [79], human MyD88 siRNA [80], human EGFR siRNA [81] or control siRNA (Dharmacon) using Lipofectamine 2000 (Invitrogen) or an Amaxa nucleofector as described [13], [15]. siRNA against mouse EGFR was synthesized using siRNA construction kit (Ambion) [82] following manufacturer's recommendation and used for mouse EGFR knock-down after transfection using Lipofectamine 2000.

### Fluorescent microscopy

To assess for LC3 accumulation around the parasite, mHEVc-LC3-EGFP cells were cultured with or without Akt inhibitor IV or transfected with either control siRNA or EGFR siRNA or treated with or without EGF (50 ng/ml; PeproTech). Monolayers were challenged with RH *T. gondii* that express cytoplasmic RFP (*T. gondii*-RFP). Five hours post-challenge, monolayers were fixed with 4% paraformaldehyde, slides were mounted using Fluoromount G and assessed for LC3-EGFP accumulation around *T. gondii* as described [13], [15]. In certain experiments, hmCD40 mHEVc-LC3-EGFP cells treated with or without CD154 were infected with WT, MIC1 ko, MIC1 ko+MIC1, MIC3 ko, MIC3 ko+MIC3, MIC1-3 ko or MIC1-3 ko+MIC1-3 tachyzoites. Monolayers were fixed with 4% paraformaldehyde, permeabilized with 0.1% Triton X-100 and incubated with rabbit anti-*T. gondii* Ab (BioGenex; San Ramon, CA) for 30 minutes. Monolayers were then washed with PBS and incubated for 1 h at room temperature with goat anti-rabbit Alexa 568-conjugated secondary antibody (Jackson ImmunoResearch Laboratories Inc., West Grove, PA). HBMEC transfected with a plasmid encoding PH-Akt-GFP were cultured with or without LY294002 followed by challenge with *T. gondii*-RFP. Distribution of PH-Akt-GFP was examined 5 min. post-challenge. In certain experiments, endothelial cells were treated with either Akt inhibitor IV or AG1478 and challenged with *T. gondii*-YFP were fixed with 4% paraformaldehyde at 8 h post-infection, permeabilized with ice-cold methanol. Monolayers were incubated overnight at 4°C with either mouse anti-human LAMP-1 or rat anti-mouse LAMP-1 (all from Developmental Studies Hybridoma Bank; Iowa City, IA). Monolayers were washed with PBS plus 1% BSA, then incubated for 1 h at room temperature with Alexa 568-conjugated secondary antibodies (Jackson ImmunoResearch Laboratories Inc.). Specificity of staining was determined by incubating monolayers with secondary antibody alone. Slides were analyzed using a Leica DMI 6000 B automated microscope equipped for epifluorescence microscopy. Experimental groups had triplicate samples and at least 100 cells per sample were counted.

### Electron microscopy

Endothelial cells were seeded onto a sterilized Aclar Embedding Film (Electron Microscopy Sciences, PA) and incubated with or without *T. gondii* tachyzoites in the presence of Akt inhibitor IV or vehicle. At 5 h post-challenge, the Aclar sheets with their attached cells were fixed as described [83]. After a soak in acidified uranyl acetate, the specimen was dehydrated in ethanol, passed through propylene oxide, and embedded in Poly/Bed (Polysciences, PA). Sections were cut in a horizontal plane parallel to that of the Aclar film to provide panoramic views of the endothelial cells. Thin sections were stained with acidified uranyl acetate in 50% methanol followed by triple lead stain of Sato. These sections were examined in a JEOL 1200 EX electron microscope (Tokyo, Japan).

### Immunoblot

Cells were lysed in buffer supplemented with protease and phosphatase inhibitors (Cell Signaling). Equal amounts of protein were subjected to either 7.5% or 10% SDS-PAGE (Bio-Rad) and transferred to PVDF membranes. Membranes were probed with either antibody to total Akt (Cell Signaling), phospho-Ser473 Akt (Cell Signaling), total EGFR (Santa Cruz Biotechnology), phospho-tyrosine 1068 EGFR (Invitrogen), phospho-tyrosine 1148 EGFR (Cell Signaling), Atg7 (Cell Signaling), Beclin 1 (BD Biosciences), PI3K p110α (Cell Signaling) or MyD88 (Cell Signaling) followed by incubation with secondary antibody conjugated to horseradish peroxidase (Santa Cruz Biotechnology). Bands were visualized by using enhanced chemiluminescence kit (Pierce Bioscience). Intensities of phospho-Akt and phospho-EGFR were calculated using ImageJ (NIH) and normalized against total Akt and total EGFR respectively.

### Statistics

Results from pooled experiments were analyzed for statistical significance using 2-tailed Student's *t* test and ANOVA (InStat version 3.0, GraphPad; La Jolla, CA). Differences were considered statistically significant when *P* was<0.05.

## Supporting Information

Figure S1 **Effects of Akt blockade on the numbers of** ***T. gondii*** **-containing vacuoles and the numbers of parasites per vacuole.** *A*, HBMEC, mHEVc and human RPE cells were incubated with or without Akt inhibitor IV (1.25 µM) for 1 h prior to challenge with *T. gondii*. In addition, HBMEC were transfected with control siRNA or Akt siRNA. Cells were then challenged with *T. gondii* 48 h after transfection. Monolayers were examined by light microscopy 24 h post-challenge to determine the numbers of parasite containing vacuoles. *B*, HBMEC were transfected with control siRNA or Akt siRNA and were then challenged with *T. gondii*. Monolayers were examined by light microscopy 24 h post-challenge to determine the numbers of parasites per vacuole. Results are shown as the mean ± SEM and are representative of at least 3 independent experiments.
(TIF)

Figure S2 **Effects of MIC8 depletion on Akt activation.** Conditional MIC8 ko parasites were cultured in HFF with or without ATc (1 µg/ml) for 48 h. Tachyzoites were harvested and incubated with HBMEC for the indicated time points. Cell lysates were used to probe for total Akt and phospho-Ser473 Akt by immunoblot. Results shown are representative of 3 independent experiments.
(TIF)
