# Supplementary figures and images for: Toxoplasma gondii-Induced Activation of EGFR Prevents Autophagy Protein-Mediated Killing of the Parasite
Source: PLoS Pathog. 2013 Dec 19;9(12):e1003809. doi: 10.1371/journal.ppat.1003809 (PMC3868508; doi:10.1371/journal.ppat.1003809)

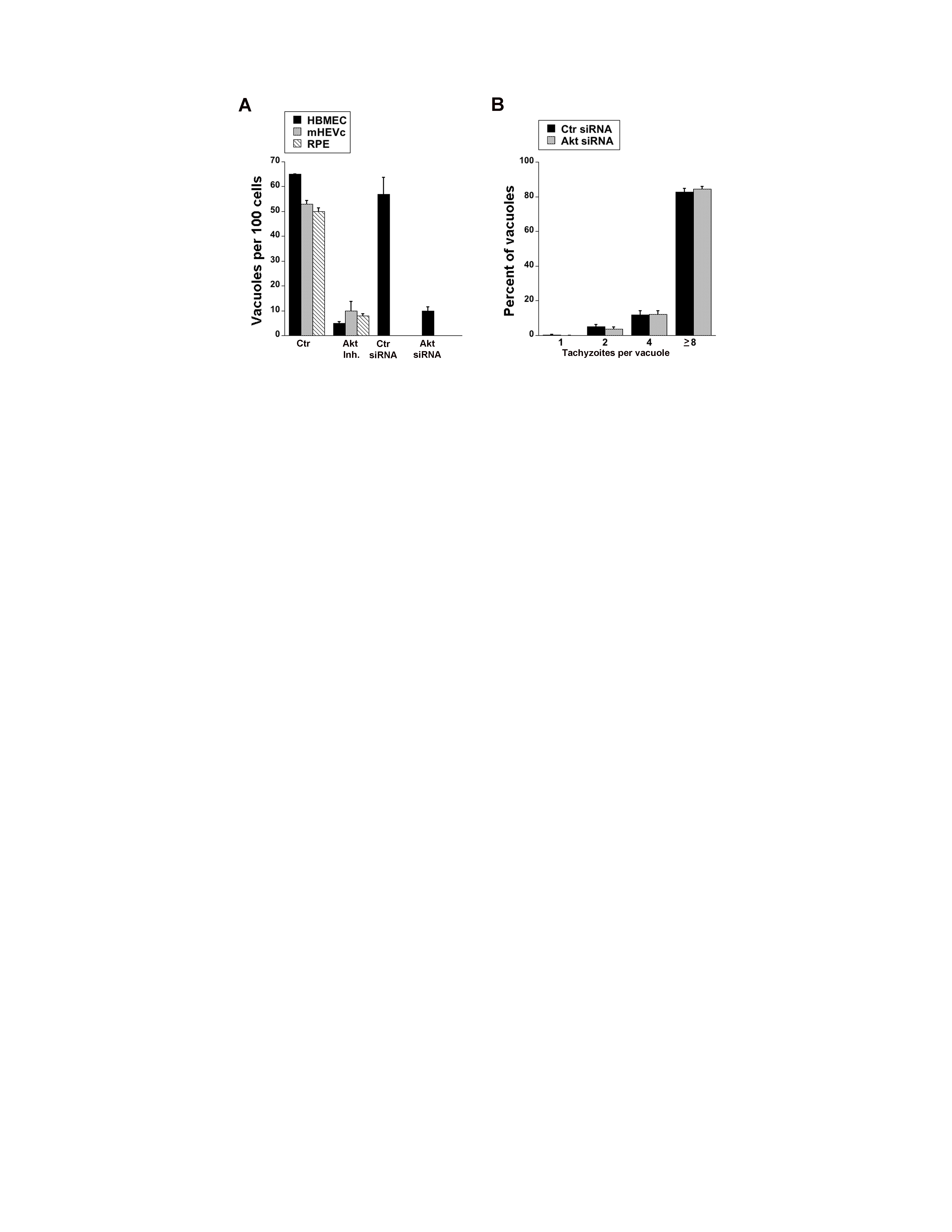

Supplement: Figure S1 — Effects of Akt blockade on the numbers of T. gondii -containing vacuoles and the numbers of parasites per vacuole. A, HBMEC, mHEVc and human RPE cells were incubated with or without Akt inhibitor IV (1.25 µM) for 1 h prior to challenge with T. gondii. In addition, HBMEC were transfected with control siRNA or Akt siRNA. Cells were then challenged with T. gondii 48 h after transfection. Monolayers were examined by light microscopy 24 h post-challenge to determine the numbers of parasite containing vacuoles. B, HBMEC were transfected with control siRNA or Akt siRNA and were then challenged with T. gondii. Monolayers were examined by light microscopy 24 h post-challenge to determine the numbers of parasites per vacuole. Results are shown as the mean ± SEM and are representative of at least 3 independent experiments. (TIF) [file ppat.1003809.s001.tif]
